# TNF Receptor Type II as an Emerging Drug Target for the Treatment of Cancer, Autoimmune Diseases, and Graft-Versus-Host Disease: Current Perspectives and *In Silico* Search for Small Molecule Binders

**DOI:** 10.3389/fimmu.2018.01382

**Published:** 2018-06-18

**Authors:** Faraz Shaikh, Jiang He, Pratiti Bhadra, Xin Chen, Shirley W. I. Siu

**Affiliations:** ^1^Department of Computer and Information Science, Faculty of Science and Technology, University of Macau, Macao, China; ^2^State Key Laboratory of Quality Research in Chinese Medicine, Institute of Chinese Medical Sciences, University of Macau, Macao, China

**Keywords:** TNF receptor type II, TNF, regulatory T cells, virtual screening, drug discovery, MM-PBSA

## Abstract

There is now compelling evidence that TNF receptor type II (TNFR2) is predominantly expressed on CD4^+^Foxp3^+^ regulatory T cells (Tregs) and myeloid-derived suppressor cells (MDSCs), and plays a major role in the expansion and function of Tregs and MDSCs. Consequently, targeting of TNFR2 by either antagonists or agonists may represent a novel strategy in the treatment of cancer and autoimmune diseases, by downregulating or upregulating suppressor cell activity. The advance in the understanding of complex structure of TNFR2 and its binding with TNF at molecular levels offers opportunity for structure-guided drug discovery. This article reviews the current evidences regarding the decisive role of TNFR2 in immunosuppressive function of Tregs and MDSCs, and the current effort to develop novel TNFR2-targeting therapeutic agents in the treatment of cancer, autoimmune diseases, and graft-versus-host disease. To shed light on the potential TNFR2-targeting small molecules, we for the first time performed virtual screening of 400,000 natural compounds against the two TNF-binding sites, regions 3 and 4, of TNFR2. Our result showed that the top hits at region 4 had slightly higher docking energies than those at region 3. Nevertheless, free energy calculation from the TNF–TNFR2 molecular dynamics simulation revealed that the binding strength of TNF in region 3 is only one-tenth of that in region 4. This suggests that region 3 is a potentially more viable binding site to be targeted by small molecules than region 4. Therefore, the effectiveness in targeting region 3 of TNFR2 deserves further investigation.

## Introduction

Tumor necrosis factor-alpha (TNF) is a pleiotropic cytokine that plays a major role in immune and inflammatory responses through two distinct receptors: TNF receptor type I (TNFR1, also known as p55 and TNFRSF1A) and TNF receptor type II (TNFR2, also known as p75 and TNFRSF1B). TNFR1 is ubiquitously expressed on almost all cell types, and TNF–TNFR1 signaling has various functions such as activation of nuclear factor kappa B (NF-κB) and induction of cell death, which depends on its cellular environment (1). By contrast, TNFR2 is more restrictedly expressed on certain cell types, such as minor subsets of lymphocytes (2, 3), endothelial cells (4), and human mesenchymal stem cells (5). Importantly, TNFR2 is predominantly expressed on the mouse and human CD4^+^Foxp3^+^ regulatory T cells (Tregs) (2), which are professional immunosuppressive cells in mammals (6). There is compelling evidence that TNFR2 expression not only defines the maximally suppressive Treg subset (2, 7) but also plays a decisive role in the proliferative expansion, suppressive function, and phenotypical stability of Tregs (8–14). TNFR2 agonist has been approved to be a novel approach for the treatment of autoimmune diseases and graft-versus-host disease (GvHD) (15), while TNFR2 antagonist has the potential to enhance antitumor immune responses (16), by upregulating or downregulating Treg activity.

Virtual screening (or *in silico* screening), the search for potential drug leads to specific target receptor by computer programs, is of central importance in early-stage drug discovery (17). In structure-based virtual screening, each compound from a large library of small molecules is docked to the ligand-binding site of the target and its binding affinity is estimated based on the predicted optimal-binding pose using an empirical scoring function. High-quality docking predictions not only reduce the time and cost for experiment but also offer in-depth structural details about the interactions of the target with ligands useful for further optimization.

Unlike TNFR1, no *in silico* studies about TNFR2 has been reported so far, and no small molecules targeting TNFR2 have been identified. Here, we aim to provide an *in silico* perspective on the potential binders to the two TNF-binding regions of TNFR2, namely, region 3 and region 4, identified from the TNF–TNFR2 structure (18). Moreover, molecular dynamics (MD) simulation combined with Molecular Mechanics-Poisson Boltzmann Surface Area (MM/PBSA) method was used to assess the per-residue energy contribution in the complex binding of key residues important to target TNFR2.

## TNFR2 Agonists Stimulate the Expansion and Activation of Tregs

Immunosuppressive Tregs are a subset of Foxp3-expressing CD4 T cells which play an indispensable role in the maintenance of immune homeostasis and prevention of autoimmune reactions (19, 20). Defect in Tregs is attributable to the pathogenesis of autoimmune diseases, such as systemic lupus erythematosus, multiple sclerosis, type 1 diabetes (T1D), rheumatoid arthritis (RA), autoimmune thyroid disease, psoriasis, inflammatory bowel disease, and autoimmune liver disease (21). Therefore, restoring the function or increasing number of Tregs has become a therapeutic strategy and the goal of treatment for patients with autoimmune diseases and GvHD (22).

We for the first time showed that TNF has the capacity to induce the activation and proliferation of Tregs (14). This effect of TNF is mediated by TNFR2, one of the TNF receptors that is predominately expressed by Tregs (2, 7, 23–25). TNFR2^+^ Tregs are the most potent suppressors, while TNFR2^−^ Tregs, even Foxp3^+^, have minimal or no suppressive activity (2, 7, 23). Furthermore, TNFR2 is also critical for the stabilization of phenotype of Tregs, in term of Foxp3 expression, and survival in the inflammatory environment (4, 9). It was shown recently that TNF priming induces the proliferation and activation of Tregs *in vivo via* TNFR2 that prolongs animal survival when compared with unprimed Tregs in acute mouse GvHD model, and TNF–TNFR2 interaction represents a novel therapy to prevent GvHD after allogeneic hematopoietic stem cell transplantation (allo-HCT) (12, 13). In a mouse model of autoimmune diabetes, TNF produced by pathogenic Teffs stimulates the expansion and suppressive function of Tregs through TNFR2 (8). In RA patients, anti-TNF therapy drives the expansion of Tregs by enhancing the binding of membrane-bounded TNF (mTNF) expressed by monocytes to TNFR2 (26). Taken together, these studies indicate that TNFR2 is an emerging target to expand functional Tregs for the treatment of autoimmune diseases and GvHD. Several agonistic TNFR2-recognizing monoclonal antibodies have been developed to expand functional Treg populations *in vitro* or *ex vivo* and showed therapeutic effects in T1D and skin inflammation (27–29). STAR2 protein, a selective mouse TNF-based agonist of TNFR2, has been shown to expand host-type radiation-resistant Tregs and improve the outcome after allo-HCT, prolong the survival without compromising the anti-leukemia or anti-infective effects in a mouse model of GvHD (11). These findings shed a light on the therapeutic potential of novel TNFR2-targeting agents in the treatment of autoimmune and inflammatory diseases. However, small molecule agonist of TNFR2 has not been identified so far.

## TNFR2 Antagonists Inhibit the Suppressive Activity of Tregs

TNFR2-expressing Tregs accumulate in the tumor microenvironment and presumably represent a major cellular mechanism of tumor immune evasion. In mouse Lewis lung carcinoma and the 4T1 breast tumor model, the majority of tumor-infiltrating Tregs have abundant surface TNFR2 expression and they are highly immunosuppressive (2, 30). In lung cancer patients and ovarian cancer patients, the proportion of TNFR2^+^ Tregs is increased in the peripheral blood or in the tumor-associated ascites (31, 32). Single-cell RNA-Seq shows that TNFR2 is one of the most markedly increased genes expressed by Tregs, when compared with CD4^+^ effector T cells (Teffs) cells and CD8^+^ cytotoxic T lymphocytes (CTLs) in metastatic melanoma patients, and the expression of TNFR2 is associated with CD8^+^ CTLs exhaustion (33). Furthermore, the expression of TNFR2 on Tregs is associated with greater lymphatic invasion, a higher incidence of tumor metastasis, a higher clinical stage, and poorer response to the treatment in patients with lung cancer and acute myeloid leukemia (31, 34, 35).

In addition to Tregs, TNFR2 is also expressed on myeloid-derived suppressor cells (MDSCs) and some tumor cells. It has been shown that mTNF, by interacting with TNFR2, activates MDSCs and enhances their suppressive activities, including upregulating arginase-1 and inducible NO synthase transcription, promoting secretion of NO, reactive oxygen species, interleukin (IL)-10, and transforming growth factor beta (21, 36). TNFR2^+^ MDSCs have the capacity to promote liver and lung metastasis of tumor (37). The signaling of TNFR2 is responsible for the accumulation and survival of MDSCs through upregulation of cellular FLICE-inhibitory protein and inhibition of caspase-8 activity (3). Moreover, TNFR2 is also expressed by tumor cells, including colon cancer (38), Hodgkin lymphoma (39), myeloma (40), renal carcinoma (41), and ovarian cancer (42). Therefore, TNFR2 is considered as an oncogene and targeting of TNFR2 with antagonistic antibodies as a novel strategy in cancer immunotherapy have been studied recently. For example, it was reported that antagonistic antibody targeting TNFR2 induces the death of both Tregs and OVCAR3 ovarian cancer cells, which have abundant surface TNFR2 expression (42). Our group found that TNFR2-blocking antibody markedly enhanced the efficacy of immunotherapy with CpG in mouse colon cancer model (43). This combination therapy resulted in the marked reduction of TNFR2 expression on tumor-infiltrating Tregs and consequently increases tumor infiltration of interferon-gamma-producing CD8^+^ CTL (44). Thus, novel antagonists against TNFR2 are potential drug candidates for cancer immunotherapy.

## Virtual Screening of Small Molecules Targeting TNFR2

Despite the important roles of TNFR2 in cancer, autoimmune diseases, and GvHD, to the best of our knowledge, no small molecule agonists or antagonists against TNFR2 have been successfully identified. With the recently available TNF–TNFR2 crystal structure (18), the specific binding pattern between TNF and TNFR2 has been revealed. This information is crucial for successful design of molecules that can directly compete against TNF to bind with TNFR2 by means of virtual screening. In virtual screening, a library of compounds is examined to predict their binding poses and binding affinities at the potential binding site of the target protein. Compounds that resemble the binding pose to the native ligand with better binding affinity will be selected as candidates for further research and development in the drug discovery pipeline. Several previous studies on virtual screening of small molecules against TNF and TNFR1 are exemplary. For example, Choi et al. screened 240,000 compounds *in silico* against the TNF dimer, and 3 compounds with a common derivative of the pyrimidine-2,4,6-trione moiety were found to be the top binders to TNF and all of them showed marked inhibitory activities in *in vitro* experiment (45). In another study, Chan et al. identified two natural product-like TNF inhibitors—quinuclidine and indoloquinolizidine—from over 20,000 compounds by virtual screening. Their activities to inhibit the binding of TNF to TNFR1 were experimentally validated. The result showed that indoloquinolizidine had the similar potency (IC_50_ = ~10 µM) as SPD304 (IC_50_ = ~3 µM), the most potent TNF binder known at that time (46). To date, the most potent small molecule antagonist targeting TNF is C87 (*K*_d_ = 0.11 µM). It was again found by virtual screening from a library of 90,000 compounds (47). In addition to virtual screening, the molecular structure of the protein–ligand complex can be used to guide the design of larger molecules such as peptides. Using the critical binding sites of TNFR1 by TNF as a template, Takasaki et al. successfully designed the first exocyclic peptidomimetics which act as TNF antagonists (48). Regarding TNFR1, using a homology model of TNF–TNFR1 complex, Chen et al. successfully found one ligand that binds to TNFR1 out of 20 hits from virtual screening of ~213,000 compounds (49); though these ligands do not show improved affinity to TNFR1 than the antagonist physcion-8-*O*-β-d-monoglucoside (*K*_d_ = 0.376 µM) identified by Cao et al. in high-throughput screening experiments (50).

The crystal structure of TNF–TNFR2 suggests that major interactions between TNF and TNFR2 occur in two regions, namely, regions 3 and 4. In region 3 of TNFR2, it contains three acidic residues, such as Asp54, Glu57, and Glu70, which together create a highly negatively charged molecular surface. On the other hand, region 4 contains three basic residues, such as Arg77, Lys108, and Arg113, which form a highly positively charged surface. Since the two centers of the binding regions are separated by a distance of at least ~20 Å, TNF binding resembles two short arms holding onto regions 3 and 4 simultaneously. To gain an insight into the relative contribution of the two regions to the overall binding, we performed a MD simulation of the TNF–TNFR2 complex [PDB 3ALQ (18)] and MM/PBSA calculation using the GROMACS simulation package (51) and the *g_mmpbsa* tool (52) to assess the free energy of protein–ligand binding. Our result shows that all three key basic residues, such as Arg77, Lys108, and Arg113, in region 4 contribute significantly to the binding energy with a total of ca. −153 kcal/mol. By contrast, the two acidic residues in region 3, such as Glu57 and Glu70, together contribute only ca. −14 kcal/mol and Asp54 did not show strong interaction with TNF. As the binding strength of TNF in region 3 is only one-tenth of that in region 4, this suggests that ligand binds in region 3 may be more competitive against TNF than in region 4. On the other hand, since region 4 is the stronger binding site for TNF, targeting region 4 with small molecules would be highly challenging, although the inhibitory effect should be greater if succeed.

As a first attempt to identify potential TNFR2 binders, we performed virtual screening of 400,000 natural compounds from the Traditional Chinese Medicine Database (53). This comprehensive natural compound library was successfully used to find potent inhibitors for EGFR (54), SIRT1 (55), and H1 (56), etc. After preparation of the TNFR2 structure by the *Preparation wizard* of Schrödinger software (57), the Glide docking box was defined to include both regions 3 and 4. The virtual screening workflow included the ligand preparation step and a pre-filtering step to screen out compounds neither satisfying the Lipinski’s rule of five nor the criteria of Absorption, Distribution, Metabolism, Elimination and Toxicity using the *QikProp* module. Filtered compounds were subjected to *Glide* high-throughput virtual screening, followed by standard precision docking and Extra Precision (XP) docking. Candidates with high XP scores and Glide energies were analyzed for their residual binding patterns. Selected compounds were further subjected to QM-polarized ligand docking (QPLD) available in the *Glide* module. We also docked a model tripeptide *RRA* which contains the same three residues of TNF that interact with TNFR2 at region 3 when bound. This is to provide a baseline energy value for compound selection.

As listed in Table 1, five top-scoring compounds for region 3 and three compounds for region 4 were obtained through this virtual screening workflow. All of them exhibited better QPLD scores (−4.624 to −6.952 kcal/mol) than the baseline molecule *RRA* (−4.404 kcal/mol). Compounds targeting region 3, the negatively charged pocket, contain amine groups that can interact with the key residues, such as Asp54, Glu57, and Glu70. However, top hits in region 4 have only slightly higher QPLD scores than top hits at region 3. Since TNF binds much stronger to region 4 than to region 3, region 4 compounds are very unlikely to be able to compete with TNF.

**Table 1:** Top-ranked compounds targeting regions 3 and 4 of TNF receptor type II from *in silico* screening.

No.	Region 3	QPLD score	Glide energy	E_vdw_	E_coul_	E_internal_	E_HB_	HB_acc_	HB_don_	Mol. weight	Rot
1	ZINC72321887	−5.366	−38.217	−10.770	−27.447	0	−3.427	7	3	316.36	10
2	ZINC67911837	−5.131	−45.119	−15.823	−29.296	14.86	−2.588	6	4	326.35	7
3	ZINC01611597	−4.518	−34.228	−4.504	−29.724	4.317	−2.700	2	4	229.31	5
4	ZINC77265363	−4.624	−44.233	−13.455	−30.778	10.036	−2.802	6	3	298.36	7
5	ZINC20465842	−4.521	−45.128	−11.514	−33.614	12.067	−2.830	4	4	281.36	8
*ref*	*RRA (baseline)*	*−4.404*	*−41.591*	*−15.455*	*−26.136*	*8.553*	*−2.924*	*14*	*12*	*456.54*	*20*

**No.**	**Region 4**	**QPLD energy**	**Glide energy**	**E_vdw_**	**E_coul_**	**E_internal_**	**E_HB_**	**HB_acc_**	**HB_don_**	**Mol. weight**	**Rot**

6	ZINC71316232	−6.952	−50.896	−27.661	−23.235	6.061	−4.911	9	5	368.34	11
7	ZINC01532677	−5.92	−31.731	−12.547	−19.184	0.000	−3.645	5	4	164.16	4
8	ZINC00281472	−5.494	−23.339	−8.581	−14.758	2.054	−1.822	6	3	222.20	5

*QPLD: QM-polarized ligand docking score; glide energy from XP docking and their energetic components: van der Waals (E_vdw_), electrostatics (E_coul_), and ligand internal energy (E_internal_); HB: energy of the hydrogen bonding term in Glide XP scoring function of the whole complex or individual key receptor residues. All energies are in kcal/mol. HB_acc_: number of hydrogen bond acceptors; HB_acc_: number of hydrogen bond donors; Mol. weight: molecular weight; Rot: number of rotatable bonds. Molecular structures of these compounds can be found in Figure S1 in Supplementary Material*.

**Figure 1:**
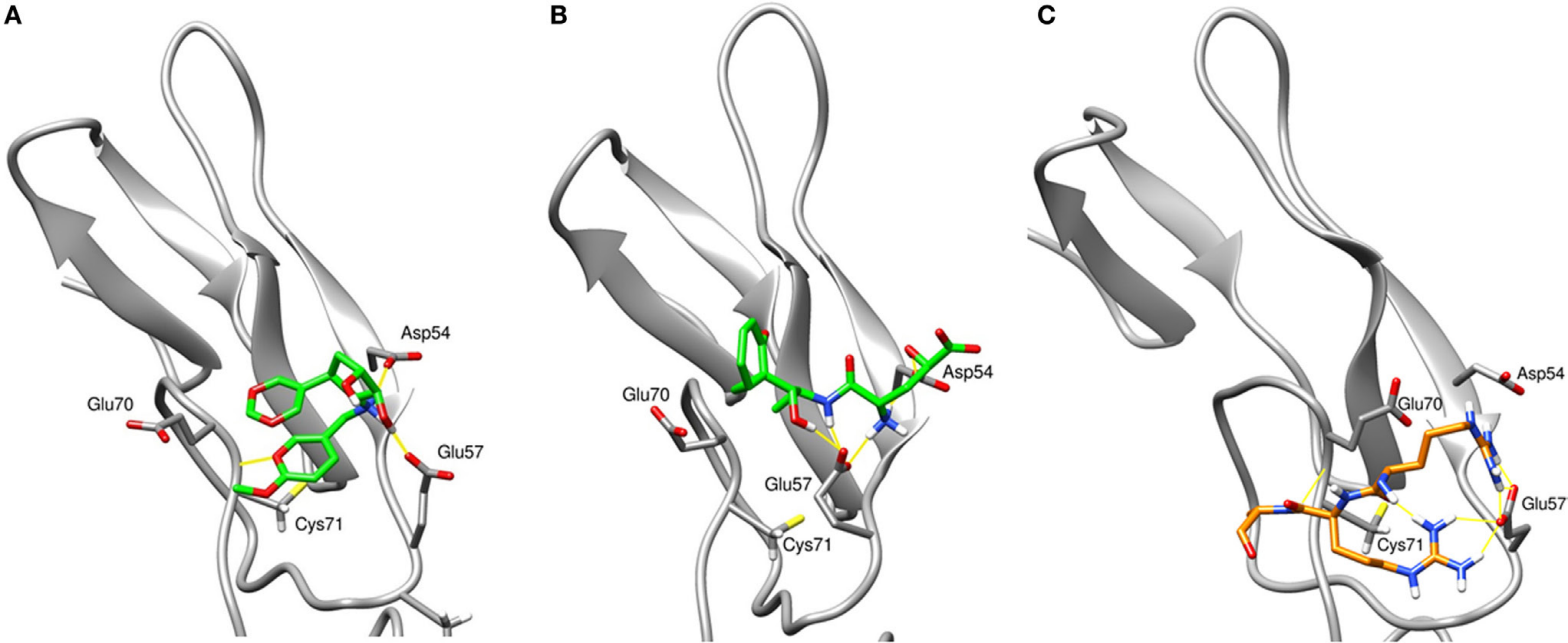
The final snapshots of 15-ns molecular dynamics (MD) simulations of the TNF receptor type II (TNFR2)–ligand complexes: **(A)** compound **ZINC72321887** and **(B)** compound **ZINC67911837** at region 3 of TNFR2. **(C)** The binding pattern of TNF–TNFR2 at the 20-ns MD snapshot. Only contacting residues, such as Arg31, Arg32, and Ala33, of TNF are displayed. The TNFR2 protein is drawn with cartoon style in gray and the ligand or TNF with sticks in green or orange. Hydrogen bonds are indicated with yellow lines.

To assess the stability of top virtual hits in region 3, compounds 1 and 2 were subjected to 15-ns MD simulations. Binding poses of these compounds are depicted in Figure 1. Compound 1 (ID ZINC72321887) is stable in the binding pocket with four hydrogen bonds. Two hydrogen bonds contributed by the hydroxyl group of the ligand that binds with Glu57 and Asp54, and the amino group with Cys71. Compound 2 (ID ZINC20465842) which contains 4 amino and 2 hydroxy groups forms 4 hydrogen bonds with Glu57 and Asp54. Our MM/PBSA analysis on the MD trajectories reveals that in the compound 1–TNFR2 complex, Asp54, Glu57, and Glu70 together contribute binding energy of ca. −70 kcal/mol in the ligand-bound state versus −13 kcal/mol in the TNF-bound state (i.e., ΔΔG = −57 kcal/mol). The enhanced binding is due to the closer contact of the ligand with Asp54 and Glu57 resulting in highly favorable electrostatic interactions and a tight network of hydrogen bonds. By contrast, in TNF–TNFR2 complex, the weak interaction of TNF with Glu70 and Asp54 is presumably caused by the flipped Glu70 side chain which pulls Arg31 of TNF to stay away from the two negative charged receptor residues. Other compounds will be further subjected to the same analysis and validated of their efficiency on inhibiting TNF-induced activation, expansion of Tregs, and enhancing antitumor immune responses in *in vitro* and *in vivo* experiments.

## Future Perspectives

Targeting TNF–TNFR2 with small molecules is a challenging task. Here, we demonstrated the use of virtual screening, MD, and MM/PBSA methods to identify promising hits from a screening of 400,000 natural compounds to target the major TNF–TNFR2 binding regions. Combined MD and MM/PBSA method can provide detail picture of the protein–protein and protein–ligand interactions which helps to identify and compare key receptor residues that contribute to the binding. Our analysis indicates that region 3 is potentially more druggable by small molecules due to its relatively much weaker but essential binding to TNF than region 4 (58). Also, TNF is not able to optimally position itself at the acidic pocket of region 3 presumably due to the physical restriction imposed by the strong binding of itself in region 4. Indeed, our top screened compound for region 3 achieved significantly better affinity (ΔΔG = −57 kcal/mol) to TNFR2 than TNF. Altogether, our study shows that the hit list targeting region 3 might serve as a good starting point to further investigate the effect of small molecules binding to TNFR2, and their efficiency on inhibiting TNF-induced activation, expansion of Tregs, and enhancing antitumor immune responses.

## Author Contributions

FS, JH, XC, and SS designed the overall project. XC supervised JH, and SS supervised FS and PB. FS performed virtual screening, FS and PB performed simulations and analyzed data. FS, JH, XC, and SS wrote and edited the manuscript. XC and SS provided funding for the project. FS and JH contributed equally.

## Conflict of Interest Statement

The authors declare that the research was conducted in the absence of any commercial or financial relationships that could be construed as a potential conflict of interest.
