# Supplementary material for: TNF Receptor Type II as an Emerging Drug Target for the Treatment of Cancer, Autoimmune Diseases, and Graft-Versus-Host Disease: Current Perspectives and In Silico Search for Small Molecule Binders
Source: Front Immunol. 2018 Jun 18;9:1382. doi: 10.3389/fimmu.2018.01382 (PMC6015900; doi:10.3389/fimmu.2018.01382)
Supplement: Supplementary file 1 [file table_1.PDF]

Supplementary Material

Perspective

TNF Receptor Type II as an Emerging Drug Target for the Treatment of Cancer, Autoimmune Diseases, and Graft-Versus-Host Disease: Current Perspectives and *In Silico* Search for Small Molecule Binders

Faraz Shaikh[1+], Jiang He[2+], Pratiti Bhadra[1], Xin Chen[2*], Shirley W. I. Siu[1*]

[1]Department of Computer and Information Science, Faculty of Science and Technology, University of Macau

[2]State Key Laboratory of Quality Research in Chinese Medicine, Institute of Chinese Medical Sciences, University of Macau

[+]Equal contribution

*Corresponding authors: xchen@umac.mo, shirleysiu@umac.mo

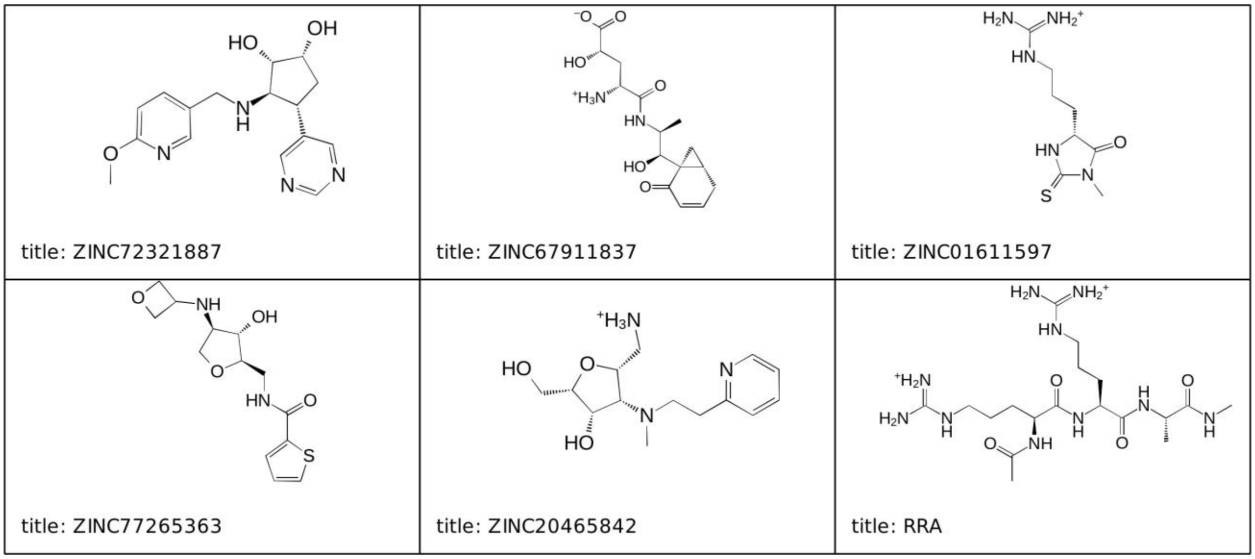

Supplementary Figure 1 (a) | TNFR2 region 3 binders. ZINC72321887:(1R,2S,3R,4R)-3-[(6-methoxy-3-pyridyl)methylamino]-4-pyrimidin-5-yl-cyclopentane-1,2-diol, **ZINC67911837:** 4-amino-2-hydroxy-5-[[1-hydroxy-1-(5-oxo-6-bicyclo[4.1.0]hept-3-enyl)propan-2-yl]amino]-5-oxopentanoic acid**, ZINC01611597:** N-[3-(1-methyl-5-oxo-2-sulfanylideneimidazolidin-4-yl)propyl]guanidine, **ZINC77265363**:N-[[(2R,3S,4R)-3-hydroxy-4-(oxetan-3-ylamino)tetrahydrofuran-2-yl]methyl]thiophene-2-carboxamide, **ZINC20465842**: [(2R,3R,4S,5S)-4-hydroxy-5-(hydroxymethyl)-3-[methyl(2-pyridin-2-ylethyl)amino]oxolan-2-yl]methylazanium, RRA: Arginine, Arginine and Alanine

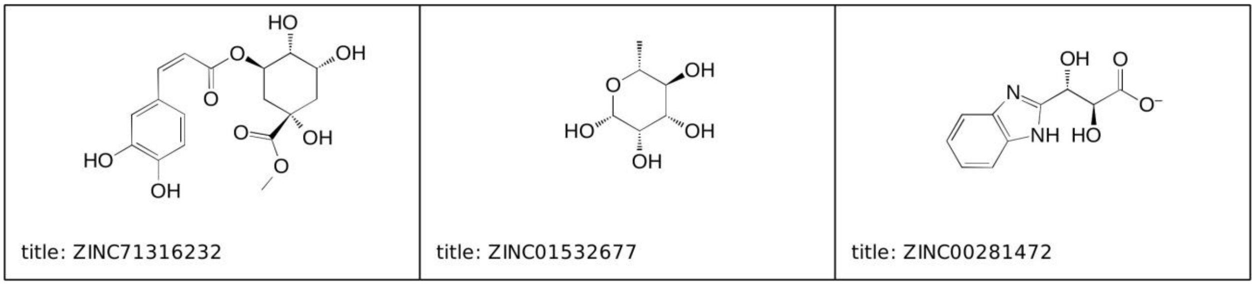

Supplementary Figure 1 (b) | TNFR2 region 4 binders. ZINC71316232: methyl (3R,4R,5R)-3-[(Z)-3-(3,4-dihydroxyphenyl)prop-2-enoyl]oxy-1,4,5-trihydroxycyclohexane-1-carboxylate, **ZINC01532677:**(2R,4S,5S,6R)-6-methyloxane-2,3,4,5-tetrol, **ZINC00281472:** (2S,3S)-3-(1H-benzimidazol-2-yl)-2,3-dihydroxypropanoic acid
